# Resolving Space-Group-Choice Dilemma in Small-Molecule Crystallography Education: A Case Study Approach

**DOI:** 10.1063/4.0000799

**Published:** 2025-10-27

**Authors:** Shao-Liang Zheng

**Affiliations:** Harvard University, Cambridge, MA, USA

## Abstract

Advancements in crystallographic equipment and software have significantly improved the accessibility of structural resolution. Despite these technological strides, the importance of teaching critical thinking skills to students remains paramount, particularly in addressing complex concepts like symmetry-related issues in crystallography. Over the last decade, we have incorporated various active learning methods and provided diverse learning opportunities to our students.[1-7] This presentation highlights our experience integrating a case-based learning approach in crystallography education to tackle space-group choice dilemmas in small-molecule crystallography.

This presentation highlights our experience integrating a case-based learning approach in crystallography education to address space-group choice dilemmas in small-molecule crystallography. Through active learning methods, we empower students to critically evaluate published crystallography data and enhance their understanding of key concepts. By gaining a solid understanding of the underlying principles behind these space-group choice dilemmas, students become more comfortable navigating different potential crystallographic solutions and independently addressing peer-review comments during their publication processes. Notably, students with well-developed critical thinking skills have subsequently contributed insights to the scientific literature based on their research experiences.[8, 10]

Our findings suggest that adopting case-based learning fosters student engagement and strengthens their analytical skills in crystallography. By sharing our insights and best practices, we aim to support educators in creating active learning environments that advance student competency in crystallography.
